# Weight Loss Promotion in Individuals with Obesity through Gut Microbiota Alterations with a Multiphase Modified Ketogenic Diet

**DOI:** 10.3390/nu15194163

**Published:** 2023-09-27

**Authors:** Hongchao Wang, Xinchen Lv, Sijia Zhao, Weiwei Yuan, Qunyan Zhou, Faizan Ahmed Sadiq, Jianxin Zhao, Wenwei Lu, Wenjun Wu

**Affiliations:** 1 State Key Laboratory of Food Science and Technology, Jiangnan University, Wuxi 214122, China; hcwang@jiangnan.edu.cn (H.W.); 6210113064@stu.jiangnan.edu.cn (X.L.); zsj960326@163.com (S.Z.); wer_yuan@163.com (W.Y.); zhaojianxin@jiangnan.edu.cn (J.Z.); 2 School of Food Science and Technology, Jiangnan University, Wuxi 214122, China; 3 Department of Nutriology, The Affiliated Wuxi People’s Hospital of Nanjing Medical University, No. 299, Qingyang Road, Wuxi 214023, China; qunyan224@163.com; 4 Flanders Research Institute for Agriculture, Fisheries and Food (ILVO), Technology & Food Sciences Unit, 9090 Melle, Belgium; faizanahmed.sadiq@ilvo.vlaanderen.be; 5 Department of Endocrinology, The Affiliated Wuxi People’s Hospital of Nanjing Medical University, No. 299, Qingyang Road, Wuxi 214023, China

**Keywords:** gut microbiome, multiphase-modified ketogenic diet, weight loss, metagenomics, machine learning

## Abstract

The occurrence of obesity and related metabolic disorders is rising, necessitating effective long-term weight management strategies. With growing interest in the potential role of gut microbes due to their association with responses to different weight loss diets, understanding the mechanisms underlying the interactions between diet, gut microbiota, and weight loss remains a challenge. This study aimed to investigate the potential impact of a multiphase dietary protocol, incorporating an improved ketogenic diet (MDP-i-KD), on weight loss and the gut microbiota. Using metagenomic sequencing, we comprehensively analyzed the taxonomic and functional composition of the gut microbiota in 13 participants before and after a 12-week MDP-i-KD intervention. The results revealed a significant reduction in BMI (9.2% weight loss) among obese participants following the MDP-i-KD intervention. Machine learning analysis identified seven key microbial species highly correlated with MDP-i-KD, with *Parabacteroides distasonis* exhibiting the highest response. Additionally, the co-occurrence network of the gut microbiota in post-weight-loss participants demonstrated a healthier state. Notably, metabolic pathways related to nucleotide biosynthesis, aromatic amino acid synthesis, and starch degradation were enriched in pre-intervention participants and positively correlated with BMI. Furthermore, species associated with obesity, such as *Blautia obeum* and *Ruminococcus torques*, played pivotal roles in regulating these metabolic activities. In conclusion, the MDP-i-KD intervention may assist in weight management by modulating the composition and metabolic functions of the gut microbiota. *Parabacteroides distasonis, Blautia obeum*, and *Ruminococcus torques* could be key targets for gut microbiota-based obesity interventions.

## 1. Introduction

Obesity is considered a global health issue that not only affects the quality of life through depression, illness, and disabilities but also reduces life expectancy because it is associated with higher all-cause mortality [1,2]. The last few decades have witnessed an alarming surge in obesity and its associated metabolic disorders. Globally, approximately two billion adults are considered overweight, of which almost half are obese [3,4]. Current approaches to obesity primarily involve lifestyle interventions [5], with dietary interventions being widely regarded as one of the most promising avenues for weight management [6]. While such interventions often yield short-term success, weight regain is almost inevitable [7]. Novel weight management strategies are still in demand.

Current research has demonstrated substantial disparities in gut microbiota composition between obese individuals and those with normal weight and that the gut microbiome plays a crucial role in host energy metabolism [8,9,10]. The human gut microbiota affects the body’s nutrient acquisition, energy expenditure, and several metabolic pathways, owing to a wide spectrum of metabolites, including bile acids, amino acids, short-chain fatty acids, indole derivatives, tryptophan, and trimethylamine N-oxide [11,12,13,14]. Furthermore, alterations in the gut microbiota can modulate the efficacy of dietary interventions, underscoring the potential benefits of fine-tuning the gut microbiota [15,16,17]. However, the extent to which different interventions impact the gut microbiota varies [7], and research investigating the intricate interplay between dietary interventions, obesity, and gut microbiota at the species level and microbial functional composition is still limited. Identifying key responsive species and critical microbial functions in promoting dietary intervention for obesity management remains a valuable avenue for further exploration. In this context, it is worth noting that metagenomic shotgun sequencing, in conjunction with bioinformatics tools, offers an enhanced means to characterize the microbiota [18]. This approach enables a more precise prediction of the biological features of microorganisms and their potential influence on host physiology [19].

In dietary interventions for obesity, low-carbohydrate or very low-carbohydrate ketogenic diets (KDs) have gained popularity in weight management [20]. These diets induce metabolic adaptations, utilizing fatty acids and ketogenic amino acids instead of carbohydrates as the primary energy source, producing ketone bodies as a molecular byproduct and resulting in ketosis [21]. Despite numerous studies demonstrating the efficacy of ketogenic diets in promoting weight loss in humans over the years [22,23,24], their underlying mechanisms are not yet fully understood [25]. Research has indicated that the ketogenic diet also modulates the subjects’ immune responses through the mediation of gut microbiota, as evidenced in a ketogenic diet intervention study involving 17 overweight or grade I obese nondiabetic men, where a significant reduction in the relative abundance of Bifidobacterium was observed following the ketogenic diet intervention [26]. Both in vitro and in vivo experiments showed that ketogenic diet-induced β-hydroxybutyrate selectively inhibited the growth of Bifidobacterium, consequently reducing intestinal proinflammatory Th17 cell levels, demonstrating a causal role of gut microbiota in mediating the host’s immune response to diet [26]. These studies provide valuable insights into the interplay among obesity, ketogenic diets, and gut microbiota, but further research is needed to expand our understanding of how different types of KD affect the gut microbiota.

Therefore, we conducted pre- and post-intervention fecal gut microbiota species-level and functional compositional analyses of the MDP-i-KD, a safer variant of the traditional ketogenic diet. We aimed to identify specific microbial species and metabolic functions closely associated with the weight reduction induced by ketogenic diet interventions. Given the intricate interplay among species within the human gut microbiota, hypothesis testing methods may not be entirely suitable for human gut microbiota research, as they assume interdependence among microbes [27]. Therefore, we employed machine learning approaches, allowing for multivariate and nonlinear analyses, to more accurately identify species crucial for obesity treatment in the MDP-i-KD intervention. Furthermore, we correlated obesity traits with specific gut metagenomic species and microbial functions to elucidate the interactions among obesity, diet, and the human gut microbiota. This study offers insights to support future microbiome-based weight management interventions, thus enabling personalized treatments to better achieve long-term weight management goals for individuals with obesity.

## 2. Materials and Methods

### 2.1. Subjects

The study protocol was approved by the hospital ethics committee (KYLKS 201806) and registered with ClinicalTrials.gov (ChiCTR180001523). Thirteen patients with obesity were recruited from The Affiliated Wuxi People’s Hospital of Nanjing Medical University. Inclusion criteria comprised: (1) age range of 18–65 years, (2) BMI ≥ 28 kg/m^2^, and (3) stable body weight over the preceding three months. All participants had signed an informed consent form. Detailed information regarding the study design, exclusion criteria, and dietary specifics has been previously reported [28].

All participants began with a 4-week hypocaloric balanced diet (HBD) as an introductory period, followed by a 12-week MDP-i-KD intervention consisting of two cycles of the same “2 + 2 + 2” dietary regimens (Supplementary Material Figure S1): two weeks of KD, followed by two weeks of a carbohydrate-based transition diet (TD), and finally two weeks of an HBD. The nutrient limits observed at each stage are listed in Supplementary Material Table S1. In addition, the registered dietitian developed a range of food choices for each diet phase for the subjects’ reference (Supplementary Material Tables S1 and S2). During the KD phase, morning urine ketone results for all participants showed that they had entered a state of ketosis.

Each subject also performed aerobic and resistance exercises. This component was described in detail in our previous study [28]. During the experiment, the dietitian monitored the patients’ dietary information, weight data, and the presence of adverse effects in the form of pictures and text reports.

### 2.2. Anthropometric Assessment and Blood Chemistry

Before and after the MDP-i-KD intervention, anthropometric assessment and blood biochemical indicator detection were carried out for obese subjects. The detailed detection indicators and details are clarified in the research of Yuan et al. [28].

### 2.3. Sample Collection

All subjects were instructed to collect fecal samples using sterile collection tubes and to collect stools once before the start of the KD intervention and once after the end of the MDP-i-KD, respectively. Collected stools were mixed with 15% glycerol and frozen at −80 °C and were used for metagenomic analysis.

### 2.4. Metagenomic Data Processing and Quality Control

Metagenome sequencing was performed on the Illumina NovaSeq 6000 platform (Illumina Inc., San Diego, CA, USA) of Shanghai Meiji Biomedical Technology Co., Ltd. (Shanghai, China) The 26 samples submitted for examination were stool samples of 13 subjects who received the MDP-i-KD at 0 and 12 weeks of intervention. The average sequencing amount of the 26 samples was 49.2 ± 4.4 (mean ± standard deviation) million reads. The preprocessing of the original sequence includes the following procedures: Trimmomatic (version 0.39) was employed to filter low-quality sequences [29]. Sequences with an average base quality score below 30 were trimmed, and sequences longer than 60 bp after filtering were retained as a quality-controlled output. Subsequently, filtered sequences were aligned to the human reference genome (Homo sapiens genome assembly GRCh38, hg38) using BWA (version 0.7.17), Samtools (version 1.9), and BEDTools (version 2.30.0), effectively removing host-origin genes from the samples [30,31,32]. After the above quality control, an average of 37.1 ± 3.8 (mean ± standard deviation) million reads were retained per sample.

### 2.5. Analysis of Gut Microbiota Species and Functions

The high-quality sequences post-quality control were subjected to taxonomic and functional annotation using MetaPhlAn3 and HUMAnN3, respectively [33]. Notably, HUMAnN3 utilized merged paired-end data for annotation and subsequently normalized the obtained counts to relative abundance values. The stratified information for individual bacterial functional contributions was derived from the normalized abundance data of metabolic pathways. Visualization of bacterial contributions to individual metabolic pathways was achieved using the humann_barplot script. Species abundance information tables were used to calculate alpha diversity metrics. Beta diversity was calculated using the Bray–Curtis metric. Linear discriminant analysis effect size (LEfSE) was used to identify bacterial taxa driving differences before and after the MDP-i-KD [34]. For the construction of the bacterial co-occurrence network, species with a relative abundance of less than 0.01% were excluded. Spearman correlation analysis was performed on bacterial correlations before and after the MDP-i-KD. Only strong correlations (r > |0.6| and *p* < 0.01) were considered and visualized by Gephi 0.9.2.

### 2.6. Machine Learning Analysis

Metagenomic species’ ability to classify two states before and after the MDP-i-KD intervention was assessed using machine learning methods. The area under the ROC curve (AUC) served as the model evaluation metric. Feature selection was performed on the original dataset using seven methods, including univariate ANOVA (f_classif, FC), mutual information estimation, and model-based approaches like Random Forest (RF), Gradient Boosting (GB), XGBoost (XGB), LightGBM (LGB), and L1 Regularization (L1) [35]. Model-based feature selection retained features with importance scores greater than 0.001.

The obtained feature subsets from each selection method were applied to individual classifiers. To mitigate random errors, we conducted five-fold cross-validation ten times and utilized the area under the curve (AUC) score as the evaluation metric to select the feature subset and corresponding model with the best classification performance. Finally, the SHapley Additive exPlanations (SHAP) package (0.42.1) was employed to calculate the SHAP value of each feature within the subset [36], revealing their importance and impact on classification.

### 2.7. Association Analysis of Gut Microbiota and Physiological Indicators

Correlation analysis between species was conducted and we identified physiological indicators of obese subjects before and after intervention. The relative abundance of bacteria species with a mean relative abundance greater than 0.1% in 20% of the samples before and after the multi-stage ketogenic diet intervention was compared with the weight, BMI, waist circumference, hip circumference, waist-to-hip ratio, body fat, body fat percentage, and internal organs of obese subjects. Spearman correlation analysis was performed on the fat area. The correlation coefficient was −0.35 < r < 0.35, *p* < 0.05.

### 2.8. Differential Analysis of Metabolic Pathways of Gut Microbiota

To identify the key gut microbiota metabolic pathways that exhibited significant changes before and after the MDP-i-KD intervention, the Wilcoxon rank-sum test was performed using the Wilcox.test function of the R language to analyze the metabolism of the gut microbiota with significant differences before and after the intervention pathway, and we calculated the Log2 Foldchange of the metabolic pathway before and after the intervention.

### 2.9. Correlation Analysis between Metabolic Pathways of Gut Microbiota and Physiological Indicators

In order to explore the metabolic pathways that were significantly changed before and after the MDP-i-KD, an association analysis between the metabolic pathways and the physiological indicators of obese subjects was carried out. Spearman correlation analysis was performed between the metabolic pathways existing in 20% of the samples before and after the intervention and the body weight, BMI, waist circumference, hip circumference, and waist-to-hip ratio of obese subjects, and the correlation coefficient was −0.4 < R < 0.4, *p* < 0.05 correlation.

### 2.10. Statistical Analysis

A statistical analysis of clinical physiological indicators was applied to measure the effect of weight loss in the MDP-i-KD and was analyzed using the paired Wilcoxon rank-sum test. Values are expressed as mean ± SD or n (%). Spearman correlations were calculated using the corr.test function (a function of the psych package in R). The Wilcoxon rank-sum test was performed using the Wilcox.test function of the R statistical package to analyze the metabolic pathways with significant differences between the gut microbiota before and after the intervention. When *p* < 0.05, the results were statistically significant.

## 3. Results

### 3.1. MDP-i-KD Changes Biochemical Measurements and Anthropometric Characteristics of Obese Subjects

Before the intervention with the ketogenic diet, the average BMI of 13 subjects (62% male and 38% female) was 31.0 ± 2.6 kg/m^2^, and the average body weight was 86.6 ± 14.7 kg. After 12 weeks of treatment, there was a significant weight loss of 8.2 ± 2.5 kg, and the average body weight of the subjects decreased by 9.4% when compared to that before the intervention. Notably, BMI (*p =* 0.0002), waist (*p =* 0.0017), hip (*p =* 0.0016), systolic blood pressure (*p =* 0.0118), blood pressure (*p =* 0.0291), AST (*p =* 0.0357), ALT (*p =* 0.0134), Triglyceride (*p =* 0.0.0081) and HbA1c (*p =* 0.0058) all showed significant decreases after the MDP-i-KD. In addition, we observed that the total cholesterol concentration did not significantly change. Before and after the intervention, the LDL cholesterol concentration did not change significantly, but the HDL cholesterol concentration increased (Table 1). The MDP-i-KD intervention did not significantly affect BUN, creatinine, albumin, UA and ALP levels (Table 1).

### 3.2. Effect of the MDP-i-KD on Gut Microbiota

**Figure 1:**
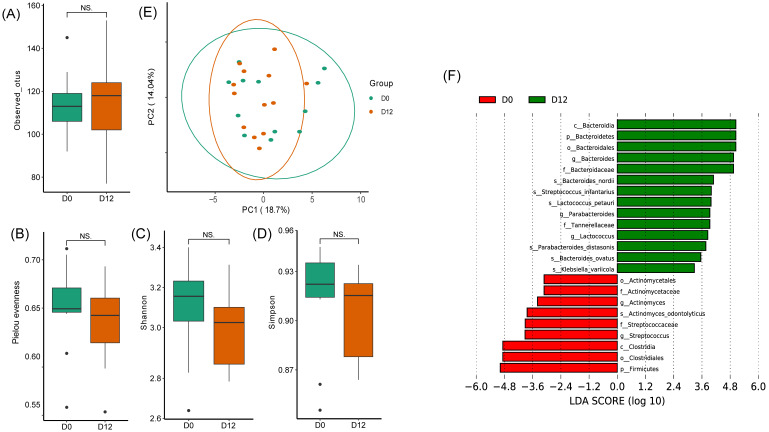
Effect of the MDP-i-KD on gut microbiota. Alpha diversity was assessed using several metrics: (**A**) observed OTUs, (**B**) Pielou’s evenness, (**C**) Shannon index, (**D**) Simpson index (NS.: *p* > 0.05). D0: before the intervention, D12: after the intervention; (**E**) Beta diversity. (**F**) LEfSe analysis identified the microbes with significant differences in abundance before and after the intervention (LDA score > 2, *p* < 0.05).

Based on the results of observed OTUs (Figure 1A), Pielou’s evenness (Figure 1B), the Shannon index (Figure 1C), and the Simpson index (Figure 1D), an assessment was made regarding the impact of the intervention on the species richness and diversity of the gut microbiota. The results indicated that none of the four diversity indices exhibited significant changes. Specifically, observed OTUs demonstrated a slight increase after the intervention, whereas the Pielou, Shannon, and Simpson indices all displayed a decreasing trend post-intervention. The increase in observed OTUs indicates a certain expansion in the variety of species within the gut microbiota after the intervention. However, the declines in the Pielou, Shannon, and Simpson indices signify reduced evenness in species distribution and decreased overall diversity in the gut microbiota. These findings suggest a potential dominance shift among certain microbial species post-intervention, resulting in heightened unevenness and decreased overall gut microbiota diversity. In addition, the beta diversity calculated by the Bray–Curtis distance did not have significant changes before and after the intervention (Figure 1E).

LEfSe analysis was used to identify six species that were significantly enriched post-intervention (D12 group): *Bacteroides nordii*, *Streptococcus infantarius*, *Lactococcus petauri*, *Parabacteroides distasonis*, *Bacteroides ovatus*, and *Klebsiella variicola*. A significant reduction in *Actinomyces odontolyticus* was observed after the intervention (Figure 1F). The above results suggest that the MDP-i-KD intervention was able to alter the gut microbiota of the participants.

### 3.3. Machine Learning Identifies Key Microbial Changes before and after MDP-i-KD Intervention

**Figure 2:**
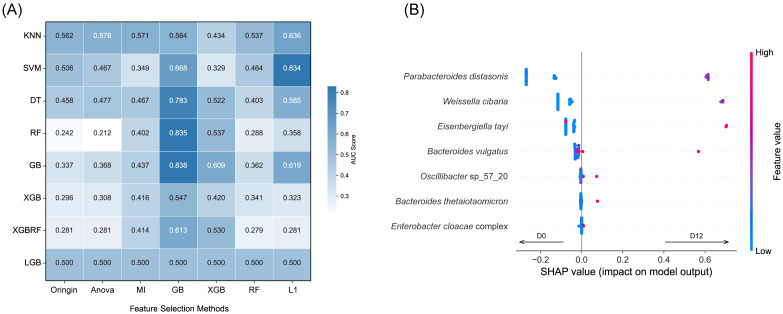
Machine learning identifies key microbial changes before and after MDP-i-KD intervention. (**A**) Impact of various feature selection methods on the performance of different models (evaluation metric is AUC). (**B**) Feature interpretation of the contribution of the seven features in the GB model to pre- and post-intervention state classification using SHAP analysis.

Machine learning analysis was conducted on metagenomic species-level data to investigate the main microbial features affected by the MDP-i-KD intervention. Seven feature selection methods were used to filter potentially redundant features for each model to reduce data dimensionality and improve classification performance. The GB and L1 methods effectively reduced features without compromising model classification performance. Specifically, the GB model identified a small subset of seven key features, including *Parabacteroides distasonis*, *Weissella cibaria*, *Eisenbergiella tayi*, *Bacteroides vulgatus*, *Oscillibacter sp*_57_20, *Bacteroides thetaiotaomicron*, and the *Enterobacter cloacae* complex. This subset achieved the best classification performance, with an AUC of 0.838 in GB (Figure 2A).

Based on the GB classification model, we ranked the relative importance of the SHAP values for the seven feature species. *P. distasonis* was identified as the most crucial species in distinguishing the pre- and post-intervention states of the MDP-i-KD. The Wilcox test confirmed a significant increase in its relative abundance after the intervention (Supplementary Material Figure S2A). Other feature species selected through the GB model did not exhibit significant changes in relative abundance before and after the intervention, indicating a low perturbation of effect values, determined by changes in their relative abundance (Supplementary Material Figure S2B–G).

Among the above characteristics, the relative abundance of *P. distasonis,* the most critical microbial feature, increased significantly after the intervention. Previous studies have identified a decrease in the abundance of *P. distasonis* in obese and metabolic syndrome populations [37,38]. Additionally, other studies of dietary interventions for treating obesity have shown an upward trend in the relative abundance of *P. distasonis* after the intervention. For example, in clinical trials with Mediterranean or low-fat, high-complex-carbohydrate diets for obesity treatment, the relative abundance of *P. distasonis* increased significantly one year after consumption [39].

Furthermore, our findings suggest an upward trend of *B. vulgatus* and *B. thetaiotaomicron* after the intervention. Recent studies have suggested a cross-feeding between *B. vulgatus* and *Akkermansia muciniphila*, positively influencing obesity reduction [40]. Additionally, other studies found a significant decrease in the abundance of *B. thetaiotaomicron* in individuals with obesity, and gavage with *B. thetaiotaomicron* protected mice from obesity [41]. These results underscore the potential importance of *B. vulgatus* and *B. thetaiotaomicron* in regulating obesity during the MDP-i-KD.

### 3.4. Species-Level Co-Occurrence Network Changes after the Intervention

To evaluate the response of microbial interactions to the MDP-i-KD, we constructed co-occurrence networks filtered using the following criteria: strong correlation (|r| > 0.6) and *p*-value < 0.01. Prior to the intervention, a total of 722 positive and 38 significant negative correlations were observed between the 299 species of participants (Table 2). Post-intervention, on the other hand, 885 positive and 16 significant negative correlations were observed between 318 species (Table 2).

**Figure 3:**
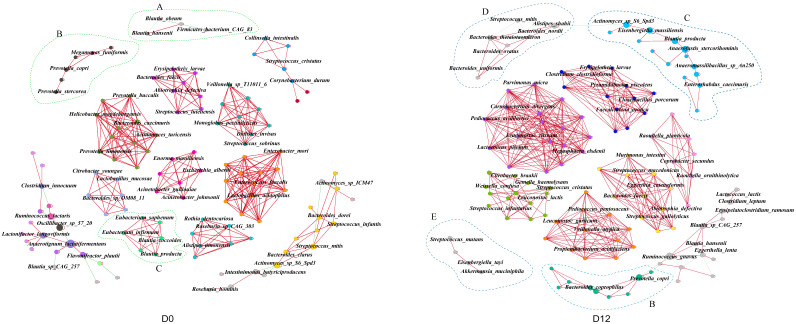
Species-level co-occurrence network changes after diet intervention. Each node was labeled by the corresponding species. A connection represents a strong (|r| > 0.6) and significant (*p*-value < 0.01) correlation. Nodes sharing the same color represent identical modules, while node size corresponds to their betweenness centrality scores. Dashed boundaries highlight alterations in interactions among obesity-related species pre-and post-intervention, (A) *Blautia obeum* module; (B) *Prevotella copri* module; (C) *Blautia producta* module; (D) *B. thetaiotaomicron* module; (E) *E. tayi* module.

We calculated the topological features of each node in the networks (Table 2). When comparisons were made between the two networks, it was found that the average clustering coefficients in the species-level co-occurrence networks increased from 0.905 to 0.920 due to the MDP-i-KD, which indicates a transition of species interactions to a more complex microbial network after the intervention. Using the modularity algorithm in the Gephi software, we clustered the closely linked nodes, and the nodes in different categories were marked differently and shown in different colors. Three major subclusters with a node count greater than ten were found in the D0 network, whereas six major subclusters with a node count greater than ten were found in the D12 network (Figure 3). While the number of modularizations decreased from 141 pre-intervention to 138 post-intervention, there was an increase in the number of subclusters containing more than 10 nodes and an overall expansion in the number of nodes within these subclusters post-intervention. These results suggest that the interactions at the species level of the gut microbiota within each subcluster are more complex and concentrated after the MDP-i-KD intervention.

In Figure 3, dashed boundaries highlight alterations in interactions among obesity-related species pre-and post-intervention. In module A, interactions involving *Blautia obeum* were lost after the intervention. Module B illustrates a negative correlation between *Prevotella copri* and *Ruminococcus gnavus*—the dominant species in the corresponding module post-intervention. In module C, *Blautia producta*, which had close associations with other species at D0, displayed disrupted interactions by D12. Conversely, two feature species, *B. thetaiotaomicron* and *E. tayi*, identified through feature selection in modules D and F, re-established their interrelationships with other species post-intervention. *B. thetaiotaomicron* exhibited a negative correlation with other anamorphic bacilli, including *Bacteroides ovatus*, *Bacteroides uniformis*, and *Baeteroides nordii*. Meanwhile, *E. tayi* showed a positive correlation with the weight loss-related species *A. muciniphila*. These findings suggest that following the MDP-i-KD intervention, subjects’ gut microbiota symbiotic network shifted toward a healthier and more intricate state.

### 3.5. MDP-i-KD Alters the Correlation between Intestinal Microbiota Abundance and Physiological Indicators

**Figure 4:**
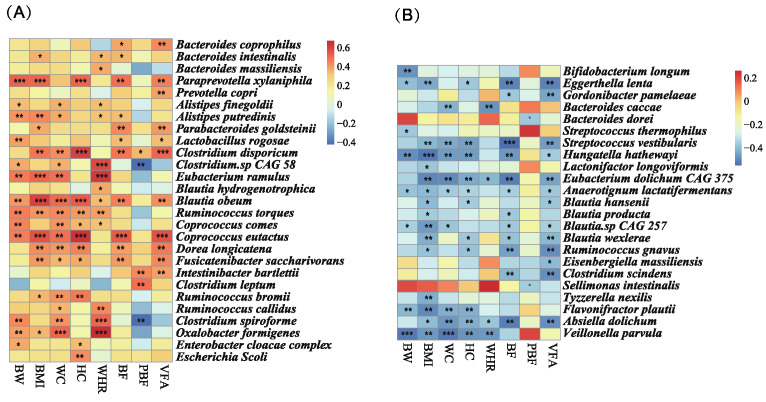
Correlation analysis of gut microbiota and physiological indicators. (**A**) Species significantly and positively linked to obesity-related physiological indicators; (**B**) Species significantly and negatively linked to obesity-related physiological indicators (*** *p* < 0.001; ** *p* < 0.01; * *p* < 0.05).

Spearman correlation analysis was performed between body weight, BMI, waist circumference, hip circumference, waist-to-hip ratio, body fat, body fat percentage, visceral fat area of obese subjects before and after the MDP-i-KD, and gut bacteria obtained by metagenomic sequencing. The results showed that *Bacteroides intestinalis*, *Paraprevotella xylaniphila*, *Alistipes putredinis*, *Parabacteroides goldsteinii*, *Clostridium disporicum*, *Eubacterium ramulus*, *Blautia obeum*, *Ruminococcus torques*, *Coprococcus eutactus*, *Dorea longicatena*, *Fusicatenibacter saccharivorans*, *R. bromii*, and *Oxalobacter formigenes* were significantly and positively associated with BMI. *P. xylaniphila*, *A. putredinis*, *C. disporicum*, *E. ramulus*, *B. obeum*, *R. torques*, *C. eutactus*, *D. longicatena*, *F. saccharivorans*, and *O. formigenes* were also significantly positively correlated with body weight, waist circumference, hip circumference, and other indicators (Figure 4). The results showed that changes in gut microbiota were closely related to the phenotypic symptoms observed in patients with obesity.

### 3.6. The MDP-i-KD Changes the Gut Microbiota Profile of the Obese Subjects

**Figure 5:**
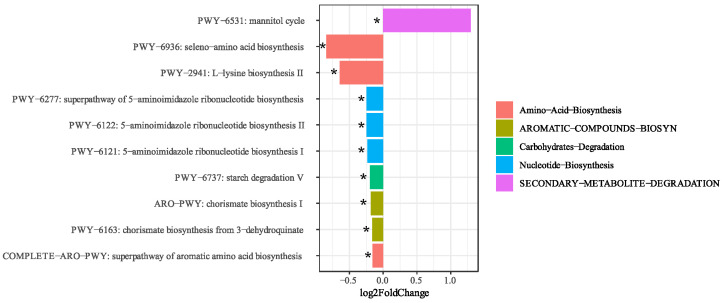
The MDP-i-KD changes the gut microbiota functional characteristics of subjects with obesity (* *p* < 0.05).

The results of the differential analysis of metabolic pathways of gut microbiota showed that before and after the multi-stage ketogenic diet intervention, the following metabolic pathways were detected: secondary metabolite degradation, amino acid biosynthesis, carbohydrate degradation, nucleotide synthesis, and aromatic compound biosynthesis of the gut microbiota in obese subjects. The results showed that ten metabolic pathways were significantly different before and after the intervention (Figure 5). The relative abundances of selenium-based amino acid biosynthesis (PWY-6936), lysine biosynthesis II (PWY-2941), the superpathway of 5-aminoimidazole ribonucleotide biosynthesis (PWY-6277), 5-aminoimidazole ribonucleotide biosynthesis II (PWY-6122), 5-aminoimidazole ribonucleotide biosynthesis I (PWY-6121), starch degradation V (PWY-6737), chorismate biosynthesis I (ARO-PWY), chorismate from 3-dehydroquinine (PWY-6163), and superchannels for aromatic amino acid biosynthesis (COMPLETE-ARO-PWY) were significantly reduced in the intestinal microbiota of post-intervention obese subjects. The relative abundance of the mannitol cycle in the gut microbiota was significantly enhanced following the intervention. These results suggest that multi-stage KD intervention alters the metabolic pathways of the gut microbiota in individuals with obesity.

### 3.7. The MDP-i-KD Alters Metabolic Pathways and Correlates with Physiological Indicators Studied

**Figure 6:**
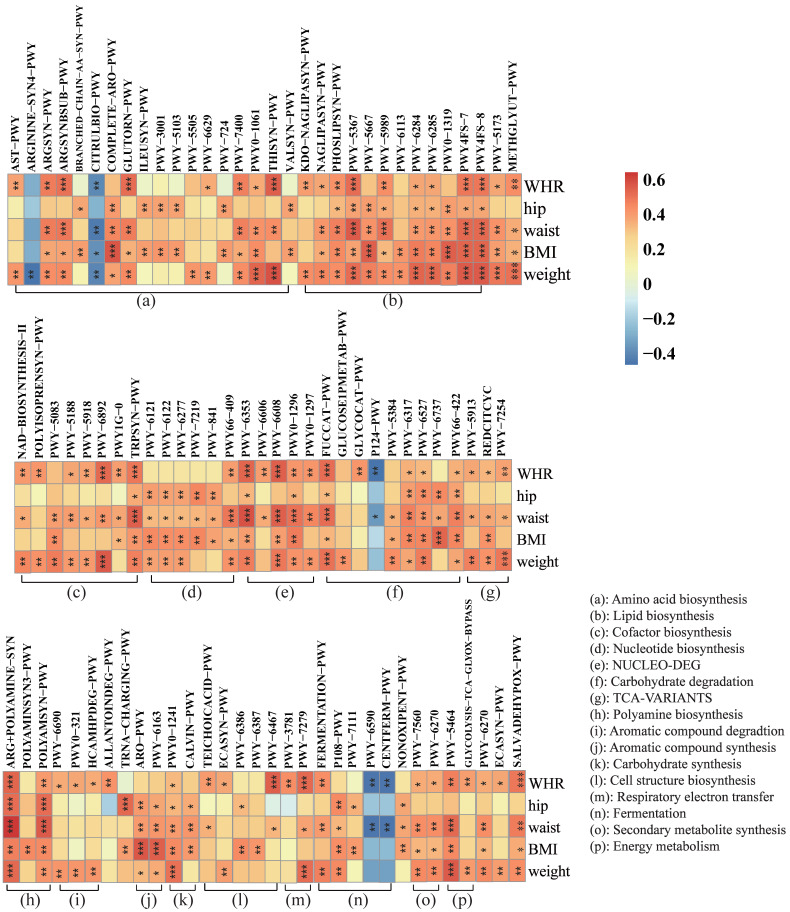
Correlation between the metabolic pathways of the gut microbiota and physiological indicators studied (*** *p* < 0.001; ** *p* < 0.01; * *p* < 0.05).

Body weight, BMI, hip circumference, waist circumference, and waist-to-hip ratio of obese subjects before and after the MDP-i-KD intervention were analyzed using Spearman correlation analysis with metabolic pathways of the gut microbiota obtained by metagenomic sequencing. The results showed that the metabolic pathway of the gut microbiome could be analyzed using the Spearman test. The changes in the subjects were closely related to their obese phenotypic symptoms (Figure 6). Including amino acid biosynthesis, lipid biosynthesis, cofactor biosynthesis, nucleotide biosynthesis, carbohydrate degradation, polyamine synthesis, aromatic compound degradation, aromatic compound biosynthesis, carbohydrate synthesis, cellular structure biosynthesis, and fermentation, a total of 93 metabolic pathways in secondary metabolite biosynthesis, energy metabolism, and other pathways were significantly correlated with obesity epigenetic indicators. Among them, 5-aminoimidazole ribonucleotide biosynthesis I (PWY-6121), 5-aminoimidazole ribonucleotide biosynthesis II (PWY-6122), 5-aminoimidazole ribonucleotide biosynthesis super pathway (PWY-6277), synthesis of chorismate from 3-dehydroquinine (PWY-6163), starch degradation V (PWY-6737), superchannel for aromatic amino acid biosynthesis (COMPLETE-ARO-PWY), and chorismate biosynthesis Synthetic I (ARO-PWY) were not only significantly positively correlated with BMI, but their relative abundance was significantly reduced after the intervention. Therefore, these seven metabolic pathways may play a vital role in reducing obesity after the MDP-i-KD intervention.

### 3.8. Species Contribution of the Metabolic Pathways of the Gut Microbiota

#### 3.8.1. Analysis of Species Contribution to Key Metabolic Pathways

**Figure 7:**
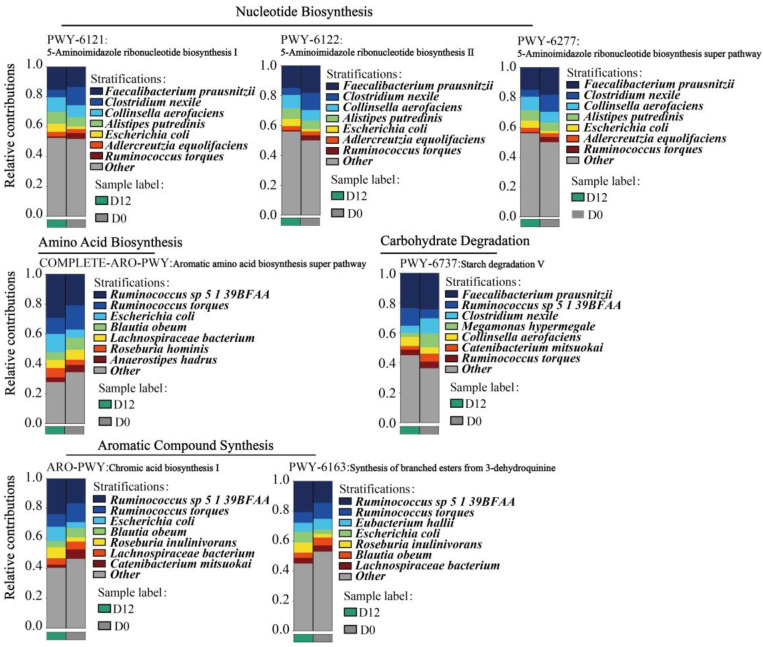
Contribution of species to key metabolic pathways.

An analysis of the metabolic pathways significantly positively correlated with BMI, with significant differences after the MDP-i-KD, including 5-aminoimidazole ribonucleotide biosynthesis I (PWY-6121), 5-aminoimidazole ribonucleotide biosynthesis I (PWY-6121), 5-aminoimidazole ribonucleotide biosynthesis II (PWY-6122), Super pathway for 5-aminoimidazole ribonucleotide biosynthesis (PWY-6277), synthesis of chorismate from 3-dehydroquinine (PWY-6163), starch degradation V (PWY-6737), superchannel for aromatic amino acid biosynthesis (COMPLETE-ARO-PWY), and chorismate biosynthesis I (ARO-PWY), was performed. Bacteria that have been reported to play an important role in these metabolic pathways were screened by calculating the contribution of species to the metabolic pathways, and the results are shown in Figure 7. *R. torques* makes a high contribution to the PWY-6121, PWY-6122, PWY-6277, ARO-PWY, PWY-6163, COMPLETE-ARO-PWY, and PWY-6737 metabolic pathways. After the MDP-i-KD and weight loss, the relative contribution of *R. torques* to key metabolic pathways was reduced, and the relative abundances of the seven key metabolic pathways were significantly reduced. Therefore, *R. torques* was significantly positively correlated with BMI, possibly by altering the key metabolic pathways in individuals with obesity. *Eubacterium hallii* contributed to a higher degree to the PWY-6163 metabolic pathway. After the intervention, the relative abundance of *E. hallii*, its relative contribution to PWY-6163, and the relative abundance of PWY-6163 significantly decreased. *B. obeum* has a high contribution to the ARO-PWY, PWY-6163, and COMPLETE-ARO-PWY metabolic pathways. The relative abundance of Blautia is highly correlated with obesity and thus diminishes with the reduction in obesity following the MDP-i-KD. *B. obeum* was significantly positively correlated with multiple obesity phenotypic indicators. After the MDP-i-KD and weight loss, the relative contributions of *B. obeum* to ARO-PWY, PWY-6163, and COMPLETE-ARO-PWY metabolic pathways decreased. We speculate that the MDP-i-KD can improve obesity symptoms by altering the relative abundance and metabolic function of *B. obeum* and *E. hallii*.

#### 3.8.2. *B. obeum* and *R. torques* Are Involved in the Regulation of Various Metabolic Activities in the Gut of Subjects with Obesity

A comparative analysis of the metabolic pathways with a relatively high contribution of *B. obeum* before and after the intervention revealed that *B. obeum* had a high relative contribution in ARO-PWY, PWY-6163, COMPLETE-ARO-PWY, and 21 other metabolic pathways (Supplementary Material Table S3). Among them, *B. obeum* can degrade formaldehyde assimilation II (hip cycle) (PWY-1861), guanosine nucleotide degradation superchannel (PWY-6595), and guanosine nucleotide degradation II (PWY-6606), which had the highest relative contribution. The analysis of the contributing pathways of *R. torques* found that in addition to the 7 key metabolic pathways, the relative contribution of bacterial species was higher, and the other 56 metabolic pathways had higher relative contributions (Supplementary Material Table S3). The above results show that *B. obeum* and *R. torques* or their metabolites can participate in a variety of metabolic activities in the guts of obese and normal-weight individuals.

## 4. Discussion

A person’s diet is considered a major determinant of the diversity, ecology, and functionality of their gut microbiota, with implications for health. Adaptation to different dietary patterns dramatically affects the onset and continuation of obesity. Although a ketogenic diet has been introduced as an effective means of weight loss, the mechanisms underlying its ameliorative effects have not yet been fully explored. Previous studies have demonstrated the potential of the ketogenic diet (KD) to modify the diversity and species composition of the gut microbiota in individuals with various health conditions, including obesity [26], type 2 diabetes [42], cancer [43], epilepsy [44,45,46], and cognitive impairment [47,48], and Alzheimer’s disease [49], as well as healthy individuals [50]. However, the majority of these investigations have predominantly centered on genus-level discussions of microbial composition, often overlooking the examination of the correlation between individual microbial species and their functional alterations in response to obesity and dietary patterns. Additionally, different types of ketogenic diets appear to exert varying effects on the gut microbiota species among subjects, highlighting the need for tailored analyses in specific contexts. In this trial, the effect of an MDP-i-KD intervention on the intestinal flora of subjects was analyzed using shotgun metagenomic sequencing. We observed significant changes in the gut microbiota composition, bacterial interaction networks, and metabolic function as a result of the multiphase dietary regimen; therefore, certain species related to weight loss were identified. Our study provides in-depth information on the effect of dietary interventions on the intestinal microbiota of individuals with obesity. We investigated not only the fecal microbiota of subjects before and after the intervention but also assessed whether any gut microbiota changes were associated with body weight loss. Therefore, our findings help clarify the interplay between obesity, diet, and the gut microbiota in human subjects.

Recent meta-analyses have shown that ketogenic diets can serve as a helpful tool for treating obesity by reducing body weight, waist circumference, and body mass index (BMI) [23]. Nonetheless, researchers recommend that a KD should be followed with caution, as its treatment of obesity may be accompanied by adverse conditions, such as dehydration, hypoproteinemia, poor adherence, and hair loss [51]. In contrast, a multi-stage dietary therapy program that includes the gradual reintroduction of carbohydrates is an efficient and safe strategy to combat obesity [52]. Therefore, the MDP-i-KD was developed in order to counteract the nutritional deficiency and low compliance of people with obesity, which may be due to KD-only interventions. It was essential to determine which species-specific alterations were caused by the MDP-i-KD intervention that led to reduced body weight. We performed shotgun metagenomic sequencing of treated feces before and after the intervention. Our results demonstrate a reduction in the alpha diversity of the gut microbiota in the intervention group, which may be attributed to the fact that carbohydrates serve as the fundamental substrate for microbial energy generation through decomposition [21]. A lower carbohydrate content in the KD could lead to an overall decrease in microbial diversity [53]. Machine learning methods identified seven crucial species associated with MDP-i-KD intervention: *P. distasonis*, *W. cibaria*, *E. tayi*, *B. vulgatus*, *O. sp*_57_20, *B. thetaiotaomicron*, and the *E. cloacae* complex. Among them, *P. distasonis* played a dominant role in distinguishing between pre-intervention and post-intervention states. Lefse analyses further confirmed a significant enrichment of *P. distasonis* post-intervention. A previous study in Mexico found that obese children and those suffering from metabolic syndrome had a reduced abundance of *P. distasonis* in their gut when compared to healthy children [37]. Similar trends were observed in a three-week intermittent fasting intervention for obesity. *P. distasonis* and *B. thetaiotaomicron* were significantly enriched after the intervention and negatively correlated with obesity-related parameters [54]. Therefore, the above findings also support the hypothesis that the decreased abundance of *P. distasonis* observed in our study may be related to metabolic changes related to weight loss after the intervention. Wang et al. demonstrated that live *P. distasonis* treatment attenuated weight gain in ob/ob and HFD-fed mice. This effect might be attributed to *P. distasonis*’s ability to convert primary bile acids into secondary bile acids (LCA and UDCA) and produce succinic acid [55]. Therefore, the above findings also support the hypothesis that the decreased abundance of *P. distasonis* observed in our study may be related to metabolic changes linked to weight loss after the intervention. Additionally, although *B. vulgatus* and *B. thetaiotaomicron* showed only an upward trend after the intervention without significant differences, they were still identified as important traits by machine learning methods, considering the complex interactions between microorganisms. *B. vulgatus* has been shown to have a cross-feeding relationship with the weight loss-associated species *A. muciniphila* [40,56,57]. Moreover, *B. vulgatus* also exhibits a cross-feeding relationship with *B. ovatus*, which we found to be significantly enriched post-intervention in the Lefse analysis [58]. As for *B. ovatus*, the trend of a childhood obesity study in Malaysia is consistent with our results, with a higher relative abundance of *B. ovatus* in the gut of normal-weight children than in obese children [59]. Moreover, *B. thetaiotaomicron* exhibited consistent results, with reduced abundance in individuals with obesity [41] and increased abundance in dietary interventions for weight loss [54]. We found that the MDP-i-KD intervention can effectively reduce *A. odontolyticus*, which causes meningitis and cervical abscess [60], indicating that the MDP-i-KD can reduce the relative abundance of harmful bacteria in the gut. These findings assure that strategies targeting the gut microbiota to treat obesity are effective. Although these differential species’ physiological function or biological behavior is not yet clear, these signatures are still worthy of attention as fine-grained gut microbiome features.

The gut microbiota is one of the most complex microbial ecosystems known in which a plethora of microbial interactions occur. Currently, most of the knowledge on the role of diet in ameliorating several metabolic disorders is limited to studying changes in the abundance of different microbial groups rather than focusing on microbial interspecific interactions [61]. Co-occurrence networks were constructed in order to search for interactions between species using metagenomic sequencing. We found significant changes in the intestinal flora co-occurrence network before and after the intervention. The co-occurrence network at the species level appeared to become more concise after the intervention, showing that the number of subclusters decreased with weight loss, which is consistent with our previous analysis of 16S rRNA. However, it is worth noting that the number of nodes in the remaining four main modules increased after the MDP-i-KD intervention, suggesting that the species-level interactions of the microbiota within each module are more complex and concentrated. Considering this, in this study, the interaction network at the microbial-species level was analyzed by metagenomic analysis, as when compared with the genus level obtained by 16S rRNA analysis, the degree of interaction between bacterial species must be more complex. In addition, pathogenic bacteria, such as pro-inflammatory and even cancer-promoting *Streptococcus infantis*, disappeared in the co-occurrence network after the intervention, suggesting they were less abundant and interacted with other species. In the D12 network, a reciprocal inhibition relationship was observed between *P. copri* and *R. gnavus*. Recent studies have indicated a significant enrichment of *R. gnavus* in insulin-resistant and obese subjects, with a notable association with low cognitive traits [62]. On the other hand, *P. copri* has been implicated in triggering host inflammatory responses and exhibits a significant correlation with adipose accumulation in pigs [63]. Several population cohort studies have indicated that *B. obeum* is associated with obesity [41]. The detailed parameters of the D12 network map show that the interaction of *B. obeum* with other species is lower than that on D0, which seems to further explain that the influence of *B. obeum* in the intestine is also reduced with weight loss. Although the interaction between microbiota species remains unclear, further studies are still needed. Overall, the above phenomena suggest that the gut flora interactions of obese participants are reorganized into a healthier state after weight loss. Our results indicate that microbial interactions in KD–obesity interactions should be considered in addition to changes in microbial abundance levels. Through co-occurrence network analysis, we have identified the impact of KD interventions on the interactions between several species that are closely associated with obesity. These species have the potential to be targeted for regulating gut microecology in obesity.

Changes in the gut microbiota are strongly associated with phenotypic symptoms in people with obesity. Previously, using AGP big data analysis, dietary interventions affecting genus-level gut microbiota, and other results, *Blautia* was consistently screened as a genus highly correlated with obesity of the gut microbiota in people with obesity. Correlation analysis between obesity phenotype indicators and intestinal bacteria showed that *Blautia obeum* was positively correlated with BMI, waist circumference, body weight, body fat, hip circumference, waist-to-hip ratio, and visceral fat area. *B. hydrogenotorophica* was significantly positively correlated with the waist-to-hip ratio. Furthermore, our findings are consistent with those of published studies. Liu et al. conducted a metagenomic association analysis and serum metabolomic analysis of obese and non-obese Chinese populations and identified a significant positive correlation between *B. obeum* and BMI [41]. Kasai et al. studied the species composition of the gut microbiota of obese and non-obese Japanese people and found that the relative abundance of *B. hydrogenotorophica* and *B. obeum* was higher in the intestines of individuals with obesity [64]. Therefore, *B. obeum* is a key bacterial species in the gut of individuals with obesity and may serve as a target for probiotics or dietary interventions for obesity. In addition, the results of the metagenomic analysis before and after intervention showed that *R. bromii*, *R. torques*, *D. longicatena*, and *E. ramulus* were significantly positively correlated with BMI. *D. longicatena* was significantly increased in the gut of the obese Chinese population in previous studies, which is consistent with our findings. *R. torques* was also confirmed to be enriched in the gut of individuals with obesity. Rosés et al. studied the effect of the Mediterranean diet on the gut microbiota of normal-weight, overweight, and obese individuals, and the results showed that the Mediterranean diet was associated with an increase in the production of butyrate and abundance of *R. bromii* [65].

We analyzed the differences seen in the metabolic pathways of the intestinal microbiota before and after the intervention and combined the correlation analysis between obesity-related physiological indicators and metabolic pathways to explain the mechanisms underlying weight loss. We observed that ten metabolic pathways were significantly altered in the intestinal microbiota of obese participants, including amino acid biosynthesis, nucleotide synthesis, secondary metabolite degradation, aromatic compound biosynthesis, and carbohydrate degradation. Among differential metabolic pathways, the relative abundance of the mannitol cycle was significantly increased after the intervention, suggesting that the ketogenic diet can promote its synthesis and metabolism. Mannitol, a known diuretic that rapidly excretes water from tissues, has not been shown to correlate with any obesity-related physiological markers. Therefore, the reasons for weight loss remain to be further explored. The graph (heatmap) shows that all nucleotide biosynthesis pathways were significantly positively correlated with BMI. Following KD intervention, a substantial decrease was observed in the relative abundance of 5-aminoimidazole ribonucleotide biosynthesis pathways I (PWY-6121) and II (PWY-6122) and the super pathway (PWY-6277). Moreover, 5-Aminoimidazole ribonucleotide is a critical intermediary in the biosynthesis of purine nucleotides, and the attenuation of its biosynthesis potentially translates into diminished purine production, thereby mitigating the accumulation of uric acid. Notably, serum uric acid levels have been positively correlated with BMI across various studies [66,67,68]. Research has observed that umami induces obesity and metabolic syndrome through the purine nucleotide degradation pathway [69]. Intriguingly, specific purine metabolites such as inosine, hypoxanthine, and uric acid have been implicated in heightened caloric intake and weight gain, even in the absence of monosodium glutamate intervention [69]. In addition, 3-dehydroquinine synthesis chorismate (PWY-6163), starch degradation V (PWY-6737), aromatic amino acid biosynthesis superchannel (COMPLETE-ARO-PWY), and chorismate biosynthesis I (ARO-PWY) were consistent with the trend in the nucleotide biosynthesis pathway, which was not only positively correlated with BMI but also significantly decreased in relative abundance after the intervention. These findings are consistent with the well-established microbial functional changes associated with obesity [13]. In a metagenomic study conducted on an obese population, it was observed that the gut microbiota in obese individuals may demonstrate elevated biosynthesis of aromatic amino acids and branched-chain amino acids, along with an enhanced capacity for carbohydrate utilization in comparison to lean controls [41]. The serum metabolomic profiles of pregnant women who had undergone malabsorptive bariatric surgery indicated reduced circulating levels of branched-chain and aromatic amino acids [70]. Additionally, a three-month supplementation with live or pasteurized *A. muciniphila* led to weight loss and the down-regulation of serum metabolomic pathways associated with tyrosine, phenylalanine, and tryptophan metabolism in obese subjects [71]. These findings suggest that the MDP-i-KD intervention may effectively diminish the relative prevalence of purine synthesis pathways, the aromatic amino acid synthesis pathway, and the starch degradation pathway within the gut microbiota, thereby curtailing the generation of purine compounds, aromatic amino acids, and their metabolites. This reduction is presumed to exert a positive influence on ameliorating obesity and metabolic syndrome. Nonetheless, comprehensive investigations are warranted to elucidate the precise mechanisms and biological ramifications of this association. It is worth noting that the contribution of these seven metabolic pathways of *R. torques* is higher, and after the multi-stage ketogenic diet intervention and weight loss, the relative contribution of *R. torques* to the seven key metabolic pathways was reduced. Therefore, *R. torques* was significantly positively correlated with BMI, possibly by altering the key metabolic pathways in individuals with obesity. *B. obeum* has a high contribution to the ARO-PWY, PWY-6163, and COMPLETE-ARO-PWY metabolic pathways, and after multi-stage ketogenic diet intervention and weight loss, the relative contribution of *B. obeum* to these three metabolic pathways was reduced. *Blautia* is a genus that is significantly associated with obesity and is significantly positively correlated with multiple obesity phenotypic indicators. Therefore, we speculated that MDP-i-KD can improve obesity symptoms by altering the relative abundance and metabolic functions of *B. obeum* and *R. torques*. As key bacteria, *B. obeum* and *R. torques* contributed to the metabolic pathway analysis results before and after the intervention, showing that they or their metabolites could participate in various metabolic activities in the intestines of obese and normal-weight individuals. Therefore, *B. obeum* and *R. torques* may be key targets for obesity intervention based on gut bacteria.

## 5. Conclusions

An increasing body of evidence suggests the relevance of the gut microbiota in the pathophysiology of diet-related metabolic disorders such as obesity. Given the intricate metabolic interactions between the host and its microbial community, the causative relationship between the gut microbiota and obesity remains elusive. Our study underscores the usefulness of the analyses of the taxa and functional groups of microbes to improve our understanding of the interactions among diet, gut microbiota, and weight loss. *Parabacteroides distasonis* exhibited the most robust response to the multiphase modified ketogenic diet (MDP-i-KD) intervention, suggesting a potential predominant role in facilitating weight loss, yet the precise mechanisms warrant further investigation. Network analysis revealed a shift in the interactions among gut microbiota toward a healthier state post-weight loss, characterized by positive associations among weight-loss-related species and inhibitory relationships among obesity-associated species after the intervention. Microbial metabolic functions indicated that the gut microbiota may promote weight loss by reducing purine compounds, aromatic amino acid generation, and carbohydrate utilization. Notably, species *B. obeum* and *R. torques*, positively correlated with BMI, contributed significantly to these pathways, with reduced relative contributions post-intervention, highlighting their potential as key targets for future obesity interventions. In summary, this work extends our previous findings [28], providing insights into future microbiome-based weight management interventions, facilitating personalized obesity treatment, and better achieving long-term weight management goals. Nevertheless, further research is required to delve into the biological mechanisms of these specific microbial signatures and their impact on obesity.

## Figures and Tables

**Table 1:** Anthropometric assessment and biochemical measurements of participants before and after the intervention.

Characteristic	Before	After	*p*-Value	Change
Body composition				
Bodyweight, kg	86.6 ± 14.7	78.4 ± 13.2	0.0017	−8.2 ± 2.5
BMI, kg/m^2^	31.0 ± 2.6	28.1 ± 2.3 *	0.0002	−2.9 ± 0.8
Waist, cm	99.3 ± 10.0	91.9 ± 8.9	0.0017	−7.3 ± 3
Hip, cm	105.4 ± 5.0	100.2 ± 5.2 *	0.0016	−5.2 ± 1.7
Blood pressure, mm Hg				
Systolic	132.3 ± 14.1	123.5 ± 14.8	0.0118	−8.8 ± 10.6
diastolic	78.4 ± 11.5	72.4 ± 11.2	0.0291	−6.0 ± 10
Liver function, U/L				
AST	26.5 ± 10.3	21.2 ± 5.8	0.0357	−5.2 ± 7.3
ALP	78.4 ± 26.4	84.0 ± 37.0	0.4214	5.6 ± 18.8
ALT	41.9 ± 30.7	27.5 ± 22.7	0.0134	−14.4 ± 19.5
Renal function				
Albumin, g/L	48.3 ± 1.3	46.6 ± 2.1	0.0645	−1.7 ± 2.5
BUN, mmol/L	4.7 ± 1.5	5.3 ± 0.8	0.0803	0.6 ± 1.5
Creatinine, μmol/L	71.5 ± 13.6	72.0 ± 13.2	0.7869	0.5 ± 5.0
UA, μmol/L	452.35 ± 29.73	438.05 ± 22.19	0.4973	−14.3 ± 37.10
Lipids, mmol/L				
Total cholesterol	5.2 ± 0.9	5.2 ± 0.9	0.6848	0.0 ± 0.8
LDL cholesterol	3.4 ± 0.8	3.4 ± 0.9	0.5292	−0.1 ± 0.7
HDL cholesterol	1.0 ± 0.2	1.1 ± 0.2	0.1259	0.1 ± 0.2
Triglyceride	2.3 ± 2.1	1.5 ± 0.7	0.0081	−0.9 ± 1.6
HbA1c [%]	5.4 ± 0.5	5.0 ± 0.4 *	0.0058	−0.4 ± 0.5

Abbreviations: BMI, body mass index; AST, aspartate transaminase; ALP, alkaline phosphatase; ALT, alanine aminotransferase; BUN, blood urea nitrogen; UA, uric acid; LDL, low-density lipoprotein; HDL, high-density lipoprotein; HbA1c, hemoglobin Alc. A Wilcoxon signed-rank test was used: * *p* < 0.05.

**Table 2:** The topological features of the network.

Variable	D0	D12
Number of edges	760.000	901.000
Number of positive edges	722.000	885.000
Number of negative edges	38.000	16.000
Number of vertices	299.000	318.000
Average degree	5.084	5.667
Average path length	2.867	2.395
Diameter	8.000	10.000
Average clustering coefficient	0.905	0.920
Centralization degree	0.036	0.028
Modularity	0.941	0.888
Number of modularity	141.000	138.000

D0: before the intervention; D12: after the intervention.

## Data Availability

Raw sequencing data have been submitted to the National Center for Biotechnology Information (NCBI) under study accession number PRJNA915838. The data presented in this study are available on GitHub: https://github.com/hcwang-jn/gut-MDP-i-KD.git (accessed on 10 August 2023).

## Supplementary Materials

The following supporting information can be downloaded at: https://www.mdpi.com/article/10.3390/nu15194163/s1, Figure S1: study design; Figure S2: Alterations in relative abundance of seven microbial features Identified by GB feature selection method pre- and post-intervention; Table S1: nutrient component of the KD, TD, and HBD; Table S2: food selection range of the KD, TD, and HBD; Table S3: gut microbiota metabolic pathways with relatively high contribution of (a) *Blautia obeum*; (b) *Ruminococcus torques*.
