# Supplementary material for: Weight Loss Promotion in Individuals with Obesity through Gut Microbiota Alterations with a Multiphase Modified Ketogenic Diet
Source: Nutrients. 2023 Sep 27;15(19):4163. doi: 10.3390/nu15194163 (PMC10574165; doi:10.3390/nu15194163)
Supplement: Supplementary file 1 [file nutrients-15-04163-s001.zip › nutrients-2586965-supplementary.pdf]

Supplementary Materials

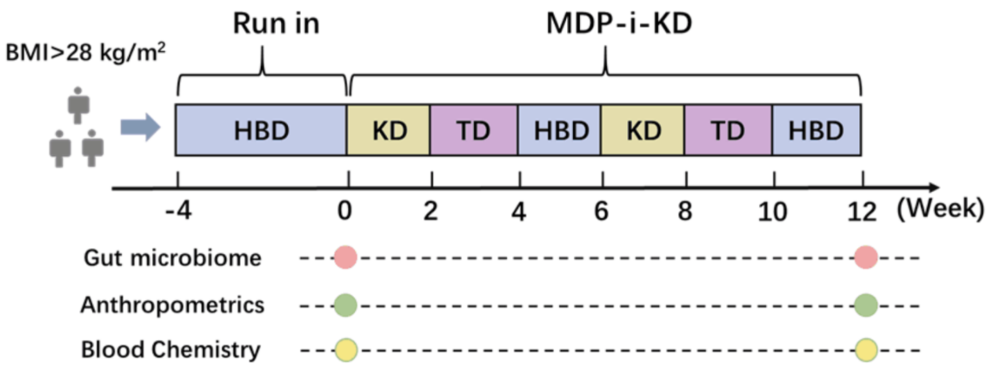

Figure S1. Study design. KD, ketogenic diet; TD, transition diet; HBD, hypocaloric balanced diet.

Table S1. Nutrient component of the KD, TD and HBD.

Nutrient component	KD[1]	TD[2]	HBD[3]
Carbohydrate, % of energy	27	29	39
Fat, % of energy	41	42	37
Protein, % of energy	32	29	24

KD[1], ketogenic diet. According to the actual measured basal metabolism of the patient's InBody S10 body composition meter, the total calories for this stage. If the basal metabolism exceeds 1500 kcal, it will be calculated as 1500 kcal. TD[2], transition diet. Multiply the basal metabolism actually measured by the patient's InBody S10 body composition meter by 1.1 to calibrate the total calories for this stage. HBD[3], hypocaloric balanced diet. Multiply the basal metabolism actually measured by the patient's InBody S10 body composition meter by 1.2 to calibrate the total calories for this stage.

Table S2. Food selection range of the KD,TD and HBD.

Food category	KD	TD	HBD
Carbohydrate	Whole grains, bran, try to choose carbohydrates with a GI value of less than 55 as the staple food	Whole grains, bran and other miscellaneous grains, try to choose carbohydrates with a GI value of less than 65 as the staple food, and add potatoes	Whole grains, potatoes, brown rice, rice noodle products, carbohydrates with a GI value of less than 65 should account for 50% of the total carbohydrates

Fat	olive oil, linseed oil	Olive oil, linseed oil, tea seed oil	Olive oil, linseed oil, tea seed oil
Lean meat	livestock lean meat, poultry lean meat	livestock lean meat, poultry lean meat	livestock lean meat, poultry lean meat
Vegetable	Tender stems, leaves, cauliflower, onion and garlic, fungus and algae	Tender stems, leaves, cauliflower, onion and garlic, fungus and algae<br>Can be added: eggplant fruit, melons and vegetables	Tender stems, leaves, cauliflower, onion and garlic, fungus and algae Can be added: eggplant, melons and vegetables, aquatic vegetables
Aquatic products	freshwater fish, freshwater shrimp	freshwater fish, freshwater shrimp<br>Can add sea fish and shrimp	Freshwater fish, freshwater shrimp<br>Can add sea fish, shrimp, shellfish
Fruit	\	Apple, kiwi, citrus	Apple, kiwi, citrus
Nut	\	Walnuts,pistachios, almonds, cashews, etc.	Walnuts,pistachios, almonds, cashews, etc.

KD, ketogenic diet; TD, transition diet; HBD, hypocaloric balanced diet; GI:Glycemic Index.

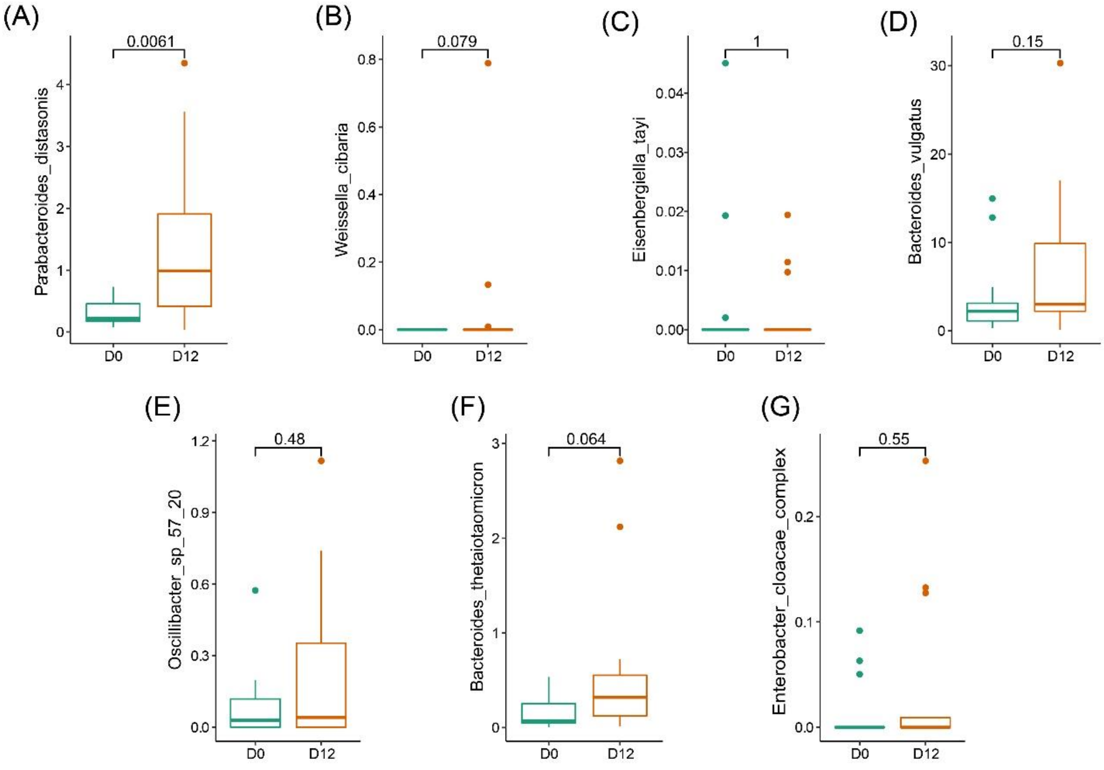

Figure S2. Alterations in the relative abundance of seven microbial features Identified by GB feature selection method pre- and post-intervention.

Table S3. Gut microbiota metabolic pathways with relatively high contribution of (a) *Blautia obeum*; (b) *Ruminococcus torques*.

(a)				
Metabolic Pathway	Function	D0 (mean)	D12 (mean)	*P* value
ARO-PWY	Branched acid biosynthesis I	18614.038	16344.379	0.039
BRANCHED-CHAIN-AA-SYN-PWY	Super channel of branched chain amino acid biosynthesis	11797.022	10511.632	0.264
COBALSYN-PWY	Recovery of cobalamine adenosine from coenzyme A amine I	5828.041	4717.161	0.505
COMPLETE-ARO-PWY	Super channel of aromatic amino acid biosynthesis	17418.475	15535.210	0.044
DTDPRHAMSYN-PWY	DTDP-L-rhamnose biosynthesis I DP-D-glucose)	9566.681	7947.129	0.418
GLYCOGENSYNTH-PWY	Glycogen biosynthesis I (from A	4404.829	3726.378	0.287
ILEUSYN-PWY	L-isoleucine biosynthesis I (from threonine)	18254.430	16585.280	0.091
NONMEVIPP-PWY	Methylerythritol phosphate pathway I	16082.643	15656.113	0.614
NONOXIPENT-PWY	Pentose phosphate pathway (non oxidative branch)	11080.130	8875.047	0.390
PWY-1861	Formaldehyde Assimilation II (Hip Circulation)	252.879	333.931	0.570
PWY-5103	L-isoleucine biosynthesisIII	10905.550	9543.020	0.264
PWY-5188	Tetrapyrrole biosynthesis I (from glutamate)	6069.769	4911.776	0.223
PWY-5189	Tetrapyrrole biosynthesis II (from glycine)	816.737	1102.869	0.920
PWY-6163	Synthesis of Branched Esters from 3-Dehydroquinine	18642.076	16558.230	0.044
PWY-621	sucrose degradation III (sucrose invertase)	8488.001	6578.999	0.186
PWY-6305	Putrescine Biosynthesis IV	3951.764	2881.691	0.762
PWY-6317	Galactose degradation I (Leloir pathway)	11624.214	8998.430	0.139
PWY-6595	A Super Channel for Guanosine Nucleotide Degradation	609.777	663.613	0.920
PWY-6606	Guanosine Nucleotide Degradation II	1020.622	1136.120	0.920
PWY-6608	guanosine nucleotide degradation III	3009.835	2974.265	0.724
PWY66-422	D-galactose degradation V (Leloir pathway)	11624.214	9107.143	0.153
THISYNARA-PWY	Thiamine diphosphate biosynthesis super channel III (eukaryote)	4834.055	4716.455	0.915
TRPSYN-PWY	L-Tryptophan Biosynthesis	6001.250	4791.738	0.186
(b)				
Metabolic Pathway	Function	D0 (mean)	D12 (mean)	*P* value

ARGSYN-PWY	L-arginine biosynthesis I (via L-ornithine)	9202.040	6763.840	0.243
ARGSYNBSUB-PWY	L-arginine biosynthesis II (acetyl cycle)	6240.492	4577.494	0.287
ARO-PWY	Branched Acid Biosynthesis I	18614.038	16344.379	0.039
COA-PWY-1	Coenzyme A Biosynthesis II (Mammals)	17248.859	16304.815	0.362
COA-PWY	Coenzyme A Biosynthesis I	6338.581	5122.028	0.687
COBALSYN-PWY	Extraction of cobalamine adenosine from cobalamide I	5828.041	4717.161	0.505
DTDPRHAMSYN-PWY	dTDP-L-rhamnose biosynthesis I	9566.681	7947.129	0.418
GLCMANNANAUT-PWY	Super pathway of N-acetylglucosamine, N-acetylmannosamine and N-acetylneuraminic acid degradation	6855.384	5677.089	0.545
GLUTORN-PWY	L-ornithine biosynthesis	4690.259	3608.188	0.336
HOMOSER-METSYN-PWY	L-Methionine Biosynthesis I	3651.106	3001.639	0.186
MET-SAM-PWY	Super pathway of S-adenosyl-L-methionine biosynthesis	5948.648	4880.070	0.169
METSYN-PWY	Biosynthesis of L-homoserine and L-methionine	5816.911	4749.004	0.169
NONMEVIPP-PWY	Methylerythritol phosphate pathway I	16082.643	15656.113	0.614
NONOXIPENT-PWY	pentose phosphate pathway (non oxidative branch)	11080.130	8875.047	0.390
PANTO-PWY	Phosphothenate Biosynthesis I	12737.325	12859.154	0.920
PANTOSYN-PWY	Pantothenic acid and coenzyme A biosynthesis I	7536.670	7164.203	0.614
PEPTIDOGLYCANSYN-PWY	peptidoglycan biosynthesis I (including m-diaminoacrylamide)	18860.404	17576.324	0.169
PWY-1042:	Glycolysis IV (plant cytoplasm)	16945.373	15459.909	0.545
PWY-2942	lysine biosynthesis III	13974.584	14096.729	0.840
PWY-5097	lysine biosynthesis VI	15130.618	15426.686	0.687
PWY-5100	Acetic acid and lactic acid production by pyruvic acid fermentation II	5675.973	4677.573	0.362
PWY-5154	L-Arginine Biosynthesis III (via N-acetyl-Lcitrulline)	2114.623	2575.627	0.687
PWY-5347	Super pathway of L-methionine biosynthesis (reverse sulfurization)	5621.599	4675.947	0.125
PWY-5367	Petroleum Arginine Biosynthesis	1059.292	767.570	0.555
PWY-5667	CDP diacylglycerol biosynthesis I	17218.189	16365.516	0.101
PWY-5686	UMP biosynthesis	19617.648	18389.549	0.287
PWY-5695	Uric acid biosynthesis/inosine 5 '-phosphate degradation	9828.490	11062.887	0.479
PWY-5973	cis vaccinate biosynthesis	6219.174	7051.029	0.311

PWY-6121	5-Aminoimidazole Ribonucleotide Biosynthesis I	17669.524	15052.789	0.022
PWY-6122	5-Aminoimidazole Ribonucleotide Biosynthesis II	17606.649	14893.396	0.022
PWY-6151	S-adenosyl-L-methionine cycle	16891.740	16135.969	0.545
PWY-6163	Synthesis of Branched Esters from 3-Dehydroquinine	18642.076	16558.230	0.044
PWY-621	sucrose degradation III (sucrose invertase)	8488.001	6578.999	0.186
PWY-6277	5-aminoimidazole ribonucleotide biosynthesis pathway	17606.649	14893.396	0.022
PWY-6305	Putrescine Biosynthesis IV	3951.764	2881.691	0.762
PWY-6317	Galactose degradation I (Leloir pathway)	11624.214	8998.430	0.139
PWY-6385	peptidoglycan biosynthesis III (Mycobacterium)	9972.206	10281.526	0.880
PWY-6386	UDP-N-acetyl-tetramyl pentapeptide biosynthesis II (containing lysine)	19958.911	18558.718	0.101
PWY-6387	UDP-N-acetyl-tetramyl pentapeptide biological synthesis I (including m-diaminotoluene)	19152.854	17828.573	0.153
PWY-6608	Guanosine nucleotide degradation III	3009.835	2974.265	0.724
PWY-6609	Adenine and adenosine	8181.517	6791.901	0.880
PWY-6700	Quioxin biosynthesis	11240.230	11988.873	0.223
PWY-6703	preQ0 biosynthesis	7403.424	8585.974	0.223
PWY-6737	starch degradation V	22301.514	19142.046	0.026
PWY-7111	Production of isobutanol by pyruvic acid fermentation (engineering)	18254.430	17099.992	0.204
PWY-7208	Hyperpathway of Pyrimidine Base	6675.339	6823.814	0.840
PWY-7219	Adenosine ribonucleotide de novo biosynthesis	25418.262	23116.118	0.223
PWY-7221	De novo Biosynthesis of Guanine Nucleoside	17568.247	16455.484	0.186
PWY-724	Super channel of lysine, threonine and methionine biosynthesis	12225.285	11372.835	0.390
PWY0-1319	CDP diacylglycerol biosynthesis II	17220.060	16368.053	0.101
PWY-7357	Thiamine and oxythiamine (yeast) form thiamine	10312.617	10071.626	0.801
PWY-7400	L-Arginine Biosynthesis IV (Archaea)	9686.627	7159.750	0.243
PWY0-1296	purine ribonucleoside degradation	15408.759	12720.948	0.091
PWY0-1297	Superpathway of purine deoxynucleoside degradation	2868.570	2614.038	0.448
PWY66-422	D-galactose degradation V (Leloir pathway)	11624.214	9107.143	0.153

RHAMCAT-PWY	L-rhamnose degradation I	3651.990	4657.016	0.287
SALVADEHYPOX-PWY	Adenosine Nucleotide Degradation II	2213.522	2501.582	0.650
SER-GLYSYN-PWY	Super channel I for L-serine and glycine biosynthesis	6599.363	5484.743	0.511
THISYN-PWY	Super channel I of thiamine diphosphate biosynthesis	1606.382	1693.235	0.724
THISYNARA-PWY	Thiamine diphosphate biosynthesis super channel III (eukaryote)	4834.055	4716.455	0.915
TRPSYN-PWY	L-Tryptophan Biosynthesis	6001.250	4791.738	0.186
VALSYN-PWY	L-valine biosynthesis	18254.430	16585.280	0.091
